# Desire for thinness among young Japanese women from the perspective of objective and subjective ideal body shape

**DOI:** 10.1038/s41598-023-41265-4

**Published:** 2023-08-29

**Authors:** Tomohiro Yasuda

**Affiliations:** School of Nursing, Seirei Christopher University, 3453, Mikatahara, Kita-Ku, Hamamatsu, Shizuoka 433-8558 Japan

**Keywords:** Ageing, Health care, Medical research

## Abstract

I examined the actual situation of the desire to be thin among young Japanese women from the perspective of ideal body shape and actual measured body shape. In total, 90 young Japanese women were evaluated using a questionnaire (perceived body shape and desired body composition change) and assessments of sarcopenia (muscle strength, physical ability, and muscle mass). Participants were classified into the underweight (body mass index [BMI] < 18.5 kg/m^2^, 74%), normal-weight (18.5 ≤ BMI < 25 kg/m^2^, 20%), or obese (25 ≤ BMI < 30 kg/m^2^, 6%) groups. The normal-weight group needed to gain an average of 2.2 kg to reach the objective ideal weight, but participants desired to lose an average of 4.5 kg. The underweight group needed to gain an average of 10.3 kg to reach the objective ideal weight, but participants desired to maintain their current body weight. Data on muscle mass for the diagnosis of sarcopenia showed low values for the underweight group. Most participants were classified into the normal-weight and underweight groups, but these groups showed a high percentage of women with a desire to be thin. The body shape of young adult women should be carefully considered not only as a health issue of thinness during the fertile period but also as a countermeasure to sarcopenia (low skeletal muscle mass) during the aging process.

## Introduction

Because the health of young adult women (in the fertile period) has a substantial impact on the health of the next generation, maintaining and improving their health is important for the health of their children, who will be the next generation. In Japan, in particular, national surveys have revealed a growing trend toward thinness among young women, and their need to increase thinness is urgent^1^. However, it has been reported that women in the fertile period in Japan have a strong desire to be thin, and there is concern about the increase in thinness and lack of exercise among pregnant women as well as low nutrition and low birth weight^2–4^. Furthermore, if thinness is not improved in approximately 21% of young Japanese women^1^, these women will be more likely to develop age-related skeletal muscle disease and muscle atrophy (sarcopenia)^5^ in the future, which is expected to affect health not only in young adulthood but also in old age.

Sarcopenia progresses rapidly after the age of 50 years and is one of the major causes of falls and nursing care in the elderly population^6,7^. Almost all sarcopenia studies have been conducted on elderly people and patients facing sarcopenia, but some recent studies have reported that approximately 30% of healthy young Japanese women already fall into the category of presarcopenia (skeletal muscle mass cutoff value only)^8,9^. Therefore, even in studies of healthy young adult women, early prevention of sarcopenia from a young age is of paramount importance.

It has been suggested that the desired body mass index (BMI) of young adult women does not always correspond to the body image that is considered healthy^10,11^, and it is important to comprehensively examine the actual state of body shape and fitness in young adult women. Therefore, in the present study, I examined the actual situation of the desire to be thin among young Japanese women from the perspective of objective and subjective ideal body shapes. Furthermore, I think it is important to focus on thinness among young adult women from the perspective of two health issues, namely, fertile period issues and future sarcopenia issues. The present study will have important implications for improving correct health awareness in young adults.

## Results

The standing height and real body weight of the participants are shown in Table 1. The present study indicated that the participants' average desired weight of 47.4 kg (mean BMI = 19.1 kg/m^2^) was significantly (4.0 kg) lower than their average measured weight of 51.4 kg (mean BMI = 20.7 kg/m^2^). Of the participants, 74%, 20%, and 6% were classified into the underweight, normal-weight, and obese groups, respectively. The obese group (mean body weight = 63.8 kg, mean BMI = 26.3 kg/m^2^) needed to lose an average of 10.4 kg to have the objective ideal weight (BMI = 22 kg/m^2^), but the participants desired to lose an average of 13.7 kg (mean BMI = 20.6 kg/m^2^). The difference between the objective ideal weight and the subjective ideal weight was statistically significant (*p* < 0.01). The normal-weight group (mean body weight = 52.2 kg, mean BMI = 21.1 kg/m^2^) needed to gain 2.2 kg to have the objective ideal weight, but the participants desired to lose an average of 4.5 kg (mean BMI = 19.3 kg/m^2^). The difference between the objective ideal weight and the subjective ideal weight was statistically significant (*p* < 0.01). The underweight group (mean body weight = 45.2 kg, mean BMI = 17.9 kg/m^2^) needed to gain an average of 10.3 kg to have the objective ideal weight, but the participants desired to gain an average of 0.4 kg (mean gain 0.4 kg, mean BMI = 18.1 kg/m^2^). The difference between the objective ideal weight and the subjective ideal weight was not statistically significant (*p* > 0.05). The ‘thinness desire index’, which I operationally defined as 100*(objective ideal BMI − subjective ideal BMI)/objective ideal BMI, showed an average of 17.8% for the underweight group, 12.3% for the normal-weight group, and 6.5% for the obese group.

**Table 1:** Physical characteristics of the participants.

Variable	Total	Underweight group	Normal-weight group	Obese group
*N*	90	18	67	5
Age	18.5 (1.0)	18.3 (0.5)	18.6 (1.1)	18.6 (0.9)
Standing height	157.4 (5.3)	158.8 (5.6)	157.2 (5.1)	155.6 (6.7)
Body weight				
Measured body weight (kg)	51.4 (6.1)	45.2 (3.7)	52.2 (4.7)	63.8 (5.4)
Subjective ideal body weight (kg)	47.4 (4.4)	45.6 (3.0)	47.7 (4.4)	50.1 (6.8)
Objective ideal body weight (kg)	54.6 (3.7)	55.5 (3.9)	54.4 (3.6)	53.4 (4.6)
BMI				
Measured BMI (kg/m^2^)	20.7 (2.2)	17.9 (0.5)	21.1 (1.4)	26.3 (0.7)
Subjective ideal BMI (kg/m^2^)	19.1 (1.3)	18.1 (0.6)	19.3 (1.3)	20.6 (1.4)
Objective ideal BMI (kg/m^2^)	22	22	22	22

Data are given mean (standard deviation). Underweight Group, BMI < 18.5 kg/m^2^. Normal-weight Group, 18.5 kg/m^2^ ≤ BMI < 25 kg/m^2^. Obese Group, 25 kg/m^2^ ≤ BMI < 30 kg/m^2^.

Subjective ideal body weight and Subjective ideal BMI, the body shape one aspires to be. Objective ideal body weight and Objective ideal BMI, the body shape objectively considered healthy.

In the obese group, all participants (100.0%) evaluated their body shape as overweight, while 52.2% and 0.0% of the participants in the normal-weight group and the underweight group evaluated their body shape as overweight, respectively. The top three desired body composition changes for the underweight group were to gain skeletal muscle (72%), lose fat (33%), and maintain body composition (17%), while the top three desired body composition changes for the normal-weight group were to lose fat (87%), gain skeletal muscle (49%), and lose skeletal muscle (9%) (Table 2).

**Table 2:** Participants' self-assessment and desire concerning body shape.

Variable	Total	Underweight group	Normal-weight group	Obese group
Perceived body shape				
Thin	4 (4.4%)	4 (22.2%)	0 (0.0%)	0 (0.0%)
Normal	46 (51.1%)	14 (77.8%)	32 (47.8%)	0 (0.0%)
Overweight	35 (44.4%)	0 (0.0%)	35 (52.2%)	5 (100%)
Desire for body composition change				
Gain fat	2 (2.2%)	2 (11.1%)	0 (0.0%)	0 (0.0%)
Gain skeletal muscle	48 (53.3%)	13 (72.2%)	33 (49.3%)	2 (40.0%)
Lose fat	69 (76.7%)	6 (33.3%)	58 (86.6%)	5 (100%)
Lose skeletal muscle	8 (8.9%)	1 (5.6%)	6 (9.0%)	1 (20.0%)
Remain body composition	5 (5.6%)	3 (16.7%)	2 (3.0%)	0 (0.0%)

Underweight Group, BMI < 18.5 kg/m^2^. Normal-weight Group, 18.5 kg/m^2^ ≤ BMI < 25 kg/m^2^. Obese Group, 25 kg/m^2^ ≤ BMI < 30 kg/m^2^.

Except for the SMI, calf girth, and SARC-CalF, there were no differences (*p* > 0.05) among the three groups in the other sarcopenia diagnostic assessments (Table 3).

**Table 3:** The assessments for AWGS 2019 in young adult females (*n* = 90).

Variable	Total	Underweight group	Normal-weight group	Obese group
Hand grip (kg)	26.5 (4.7)	25.6 (5.3)	26.8 (4.7)	25.6 (3.2)
SMI (kg/m^2^)	5.95 (0.48)	5.51 (0.37)	6.00 (0.38)**	6.83 (0.45)**^,##^
Calf girth (cm)	34.5 (2.1)	32.4 (1.3)	34.8 (1.5)**	38.8 (0.7)**^,##^
SARC-F (unit)	0.5 (0.8)	0.6 (0.9)	0.5 (0.7)	1.0 (0.8)
SARC-Calf (unit)	3.1 (4.6)	8.4 (4.6)	1.6 (3.4)**	1.0 (0.8)*
Gait speed (m/s)	1.44 (0.3)	1.45 (0.3)	1.43 (0.3)	1.47 (0.22)
5-time chair stand test (s)	5.8 (1.1)	5.9 (1.0)	5.7 (1.1)	6.5 (1.6)
SPPB (unit)	12.0 (0.0)	12.0 (0.0)	12.0 (0.0)	12.0 (0.0)

Data are given mean (standard deviation). BMI, Body mass index. Underweight Group, BMI < 18.5 kg/m^2^. Normal-weight Group, 18.5 kg/m^2^ ≤ BMI < 25 kg/m^2^. Obese Group, 25 kg/m^2^ ≤ BMI < 30 kg/m^2^. SMI, skeletal muscle index. **p < 0.01, vs. Underweight Group. *p < 0.05, vs. Underweight Group. ^##^p < 0.01, vs. Normal-weight Group.

## Discussion

The present study indicated that the participants' average desired weight of 47.4 kg (mean BMI = 19.1 kg/m^2^) was significantly (4.0 kg) lower than their average measured weight of 51.4 kg (mean BMI = 20.7 kg/m^2^), which confirmed that young Japanese women have a strong desire to be thin. The mean BMI of 20.7 kg/m^2^ was almost the same as that reported for young Japanese women in previous studies (mean BMI = 20.4 and 20.9 kg/m^2^)^12,13^. In addition, the standing height, weight, BMI, and grip strength (females: 157.4 cm, 51.4 kg, 20.7 kg/m^2^, and 26.5 kg, respectively) in the present study were similar to the Japanese reference values for morphology and physical fitness (females: 158.7 cm, 52.2 kg, 20.7 kg/m^2^, and 28.4 kg, respectively [age of 19 years])^14^. Thus, the participants recruited for this study were representative of the general population of healthy young adults in Japan at present.

The Japan Society for the Study of Obesity indicates that a BMI of 22 kg/m^2^ is an appropriate (healthy) weight for Japanese individuals^15,16^, and based on this, the average objective ideal body weight for the population in the present study (*n* = 90) was calculated to be 54.6 kg (Table 1). Therefore, the participants in the present study would need to gain an average of 3.2 kg to reach the objective ideal body weight. However, based on the results of the questionnaire survey in the present study, the participants desired to lose an average of 4.0 kg (i.e., 51.4 minus 47.4 kg) of their body weight (Table 1), which is similar to the reports of several previous studies of young Japanese women^11,17^. In the present study, the normal-weight group desired to lose an average of 4.5 kg (BMI = average 19.3 kg/m^2^), while they needed to gain an average of 2.2 kg to achieve the objective ideal body shape (BMI = 22 kg/m^2^). Furthermore, participants in the underweight group desired to maintain their present body shape (BMI = average of 18.1 kg/m^2^) even though they were already thin, with a BMI of 17.9 kg/m^2^. A previous study reported that when comparing BMIs for men and women in developed countries after adjusting for age, individuals in Asian countries tend to have lower BMIs, particularly Japanese women, who have very low values^18^. Thus, other studies have confirmed that women are significantly more dissatisfied with their body shape than men and that Japanese women have a particularly strong desire to be thin compared to women in other countries (German^19^ and Taiwanese^12^ women). Moreover, the age-specific BMI of Japanese women has continued to show a further downward trend in recent years, suggesting that Japanese women are still suffering from a lack of awareness of the current thinness crisis^20^.

A previous study^11^ of Japanese female students (12–15 years old, average BMI = 21.1 kg/m^2^) showed that most Japanese girls estimated their body shape as overweight and were dissatisfied with it, with 78.3% of the students desiring to lose weight. Therefore, it was suggested that the greatest problem related to body image is the excessive obsession with thinness among Japanese girls. As in the case of male and female university students in other Asian countries, young Japanese women are extremely dissatisfied with their body shape and have a strong desire to be thin^12,13^. Japanese women’s desire to be thin represents one of the most prominent ethnicity traits. This may be based on the fact that, for the past 40 years or more, as in developed Western countries, slim bodies have become increasingly favored in Japan as a symbol of beauty and success for young women. It has also been suggested that this may be due to advertisements, fashion and movies that have created an awareness of feminine ideals among young women in these countries^21,22^. Hence, it is likely that with the rapid economic growth, Japanese women became more rigid in their desire for physical slenderness and became dissatisfied with their own body shapes and body parts. A previous study^23^ of 960 country-years and 9.1 million participants indicated that the average BMI of Japanese women was closer to that of women in low-income countries than to that of women in most high-income countries, indicating a very serious health problem. The underweight group in the present study (average BMI = 17.9 kg/m^2^, average body fat = 21.9%) had strong misconceptions about body image, including body composition (skeletal muscle mass, fat mass, etc.), and 33% of the participants in this group desired to lose fat even though they did not need to lose weight. Unless their subjective ideal body images are corrected, instructing young women to aim for their objective ideal body weight will not be effective.

Previous studies^9,24^ have shown that a certain number of young adult women already fall into the category of presarcopenia (sarcopenia diagnostic level of skeletal muscle mass only). Furthermore, the participants in the underweight group in this study had a lower SMI, calf girth, and SARC-CalF than participants in the other groups according to the diagnostic criteria for sarcopenia (Table 3). Similar to these indices, skeletal muscle mass was measured, consistent with the results of previous studies of young Japanese women^9,24^. Therefore, young women need to improve their skeletal muscle mass to prevent the need for future nursing care. However, in the present study, 77.8% of the participants in the underweight group perceived their body weight to be normal, which means that they had a false image concerning a healthy body shape. For young women to acquire correct knowledge about a healthy body shape, the media strategy should focus on (i) changing the current misconception that a slim body is a symbol of beauty and success and (ii) disseminating information regarding a sarcopenia diagnosis at a young age, making young women aware of the importance of maintaining skeletal muscle mass and care prevention.

The present study had a limitation. The sample size of the obese group was small because young Japanese women tend to be thinner than expected. Additional studies with larger sample sizes and robust experimental designs should be performed in the future to verify the present findings.

In conclusion, the percentage of individuals who desired to be thin was high even in the normal-weight and underweight groups, to which most of the young adult women participating in this study were categorized. Thus, young adult women should be reminded of the need to increase their skeletal muscle mass. The body weight of young adult women should be carefully considered not only as a health issue of thinness during the fertile period but also as a countermeasure to sarcopenia (low skeletal muscle mass) during the aging process. It is important for young adults to be aware that low skeletal muscle mass is a condition that is likely to lead to increased sarcopenia risk in the future. In the future, it will be necessary to further improve the environment (schools, families, and social education) to prevent an extreme desire to be thin and dieting among young adult women.

## Methods

### Participants

**Figure 1:**
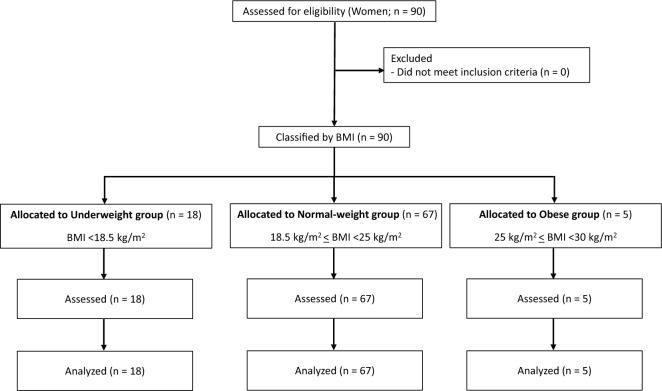
The flow diagram of the study sample.

A verbal invitation to participate in the present study was given to university students, and 90 Japanese women between the ages of 18 and 22 years were enrolled. No participant had a history of musculoskeletal disease or knee surgery. All participants were free of obvious chronic diseases (e.g., angina, myocardial infarction, diabetes, cancer, and stroke) as assessed by an annual physical examination. Before obtaining informed consent, a document explaining the purpose and safety of the study as well as a lifestyle questionnaire were handed out to potential participants. Participants in the present study were categorized as recreationally active, and 58 of the 90 participants regularly performed aerobic-type exercises (walking, jogging, and cycling for approximately 30 min 2–3 times per week). All participants who met the criteria were included in the data analysis. Participants were divided into the following three groups according to body mass index (BMI): the underweight group (BMI < 18.5 kg/m^2^), normal-weight group (18.5 kg/m^2^ ≤ BMI < 25 kg/m^2^), and obese group (25 kg/m^2^ ≤ BMI < 30 kg/m^2^)^1^ (Fig. 1). The ideal body weight was estimated from the following formula recommended by the precepts of the Japan Diabetes Society (JDS) and the Japan Society for the Study of Obesity (JASSO): ideal body weight (kg) = standing height (m) × standing height (m) × 22 (BMI, kg/m^2^). In accordance with the precepts of the JDS and JASSO, a BMI of 22 kg/m^2^ was considered ideal for Japanese individuals. The principles of the Medical Association Declaration of Helsinki guidelines for the use of human participants were adopted for this study. This research was approved by the Ethics Committee of Seirei Christopher University (approval number: 22007), and written informed consent was obtained from all participants.

### Sarcopenia assessment

Standing height was measured in 0.5-cm increments using a standing height scale, and body weight was measured in 0.1-kg increments using an electronic scale. Body mass index (BMI) was calculated as body mass/height^2^ (kg/m^2^). The multifrequency bioelectrical impedance analyzer (BIA), InBody 430 analyzer (Biospace, Seoul, Korea), was applied in accordance with the manufacturer's guidelines. The BIA estimates body composition by the differences in conductivity, which are based on the different biological properties of each tissue. This body composition analyzer is based on a 4-pole, 8-point contact electrode system, and it measures the impedance of the arms, trunk, and legs separately for each segment at three different frequencies (5, 50, and 250 kHz). Participants were measured in a resting position and standing with arms forward and elbows extended. The InBody analyzer automatically calculated body weight, total body fat percentage, and total skeletal mass as well as body fat percentage and skeletal muscle mass for each body region. The skeletal muscle index (SMI; AMM/height^2^, kg/m^2^) was calculated by summing the values of the two upper and two lower extremities (AMM)^8,25,26^.

Maximum voluntary isometric contraction (MVIC) of hand grip strength was assessed using a factory-calibrated hand dynamometer (TKK 5401; Takei, Tokyo, Japan). All participants were instructed to grasp the dynamometer in their right hand with their arms positioned beside their trunk in an upright posture and elbows extended at 180°. The dynamometer handle was set to a size that was comfortable for the participant to hold (fits the second joint of the fingers). Every participant performed two attempts, and the highest value of each attempt was used in the analysis^25^.

The normal walking test was performed on a 6-m course^8,25^.

Calf girth was measured at the widest part of both calves using an inelastic tape and recommended protocols with moderate to high sensitivity and specificity in predicting sarcopenia and low skeletal muscle mass. The AWGS 2019 criteria recommend a calf girth cutoff of less than 34 cm for men and 33 cm for women for screening and case finding^26^.

SPPB was performed on participants using the National Institute on Aging protocol. The tests were performed in the following order: (a) the standing balance test, (b) the walking test, and (c) the standing chair test. In the standing balance test, participants were instructed to maintain their posture with their feet in lateral, semitandem, and tandem directions for 10 s. Scores were set to range from 0 to 4 (maximum performance). A walking test was performed to assess the time the participants needed to walk 4 m at a standard pace. A standard stable wooden chair (0.40 m high and 0.30 m deep) was utilized to measure the time for five consecutive chair stand tests from a sitting position with both arms resting on the chest. Scores in each category ranged from 0 to 4. The walking and chair stand-up tests were based on temporal quartiles established previously in a large population; the total of the three components was the final SPPB score with a range of 0 to 12 points, and 12 points indicated the most advanced lower limb function^27,28^.

In this study, the AWGS 2019 sarcopenia diagnostic criteria for women (hand grip strength, the SMI, calf girth, SARC-F, SARC-Calf, gait speed, 5-time chair stand test, and SPPB)^28^ were applied (Table 3).

### Questionnaire

The primary outcome assessed was the degree of discrepancy between objective and subjective ideal body shapes in each individual. Participants were asked to answer the following three questions: (1) “What is the ideal body weight for you?”; (2) “How would you classify your body shape? (Please choose one of the following 3 options: thin, normal, or fat); and (3) “What body composition changes do you desire? (Please choose all that applies from the following 5 options: gain fat, gain skeletal muscle, lose fat, lose skeletal muscle or maintain body composition). Question (1) was asked to reveal the difference between the subjective and objective ideal body weight/BMI, of which the latter were specified by the WHO and obesity societies assessment criteria. Question (2) was asked to clarify the participants' perceptions of their own body shape, while Question (3) was asked to understand the participants' desired changes in body composition.

### Statistical analyses

The results are presented as the mean ± standard deviation for all variables. All data were analyzed using JMP software (ver. 12.0 SAS Institute Inc., Tokyo, Japan). The subjective ideal BMI was defined as the subjective ideal body weight (kg) divided by the standing height squared (m^2^), and the objective ideal BMI was defined as 22 kg/m^2^. The thinness desire index (%) was calculated as the objective ideal BMI − the subjective ideal BMI)/the objective ideal BMI × 100. When data were normally distributed, comparisons among the underweight, normal-weight, and obese groups were performed for sarcopenia assessments (hand grip strength, the SMI, calf girth, SARC-F, SARC-Calf, gait speed, 5-time chair stand test, and SPPB) with an unpaired t test. The Wilcoxon signed rank test was applied to identify differences in SARC-Calf among the three groups when the data were not normally distributed. Statistical significance was defined as p < 0.05.

## Data Availability

The data that support the findings of this study are not publicly available but can be obtained from the corresponding author upon reasonable request.
